# Breast Cancer Physical Activity Mobile Intervention: Early Findings From a User Experience and Acceptability Mixed Methods Study

**DOI:** 10.2196/32354

**Published:** 2022-06-22

**Authors:** Gabriel Ruiz Signorelli, Francisco Monteiro-Guerra, Octavio Rivera-Romero, Francisco J Núñez-Benjumea, Luis Fernández-Luque

**Affiliations:** 1 Adhera Health, Inc Palo Alto, CA United States; 2 The Insight Centre for Data Analytics School of Public Health, Physiotherapy and Sports Science University College Dublin Dublin Ireland; 3 Department of Electronic Technology Universidad de Sevilla Seville Spain; 4 Innovation Unit Virgen Macarena University Hospital Andalusian Health Service Seville Spain

**Keywords:** breast cancer, BC, mobile app, physical activity, mHealth, acceptability, user experience, mobile phone

## Abstract

**Background:**

Physical activity (PA) is the most well-established lifestyle factor associated with breast cancer (BC) survival. Even women with advanced BC may benefit from moderate PA. However, most BC symptoms and treatment side effects are barriers to PA. Mobile health coaching systems can implement functionalities and features based on behavioral change theories to promote healthier behaviors. However, to increase its acceptability among women with BC, it is essential that these digital persuasive systems are designed considering their contextual characteristics, needs, and preferences.

**Objective:**

This study aimed to examine the potential acceptability and feasibility of a mobile-based intervention to promote PA in patients with BC; assess usability and other aspects of the user experience; and identify key considerations and aspects for future improvements, which may help increase and sustain acceptability and engagement.

**Methods:**

A mixed methods case series evaluation of usability and acceptability was conducted in this study. The study comprised 3 sessions: initial, home, and final sessions. Two standardized scales were used: the Satisfaction with Life Scale and the International Physical Activity Questionnaire–Short Form. Participants were asked to use the app at home for approximately 2 weeks. App use and PA data were collected from the app and stored on a secure server during this period. In the final session, the participants filled in 2 app evaluation scales and took part in a short individual interview. They also completed the System Usability Scale and the user version of the Mobile App Rating Scale. Participants were provided with a waist pocket, wired in-ear headphones, and a smartphone. They also received printed instructions. A content analysis of the qualitative data collected in the interviews was conducted iteratively, ensuring that no critical information was overlooked.

**Results:**

The International Physical Activity Questionnaire–Short Form found that all participants (n=4) were moderately active; however, half of them did not reach the recommended levels in the guidelines. System Usability Scale scores were all >70 out of 100 (72.5, 77.5, 95, and 80), whereas the overall user version of the Mobile App Rating Scale scores were 4, 4.3, 4.4, and 3.6 out of 5. The app was perceived to be nice, user-friendly, straightforward, and easy to understand. Recognition of achievements, the possibility of checking activity history, and the rescheduling option were positively highlighted. Technical difficulties with system data collection, particularly with the miscount of steps, could make users feel frustrated. The participants suggested improvements and indicated that the app has the potential to work well for survivors of BC.

**Conclusions:**

Early results presented in this study point to the potential of this tool concept to provide a friendly and satisfying coaching experience to users, which may help improve PA adherence in survivors of BC.

## Introduction

### Background

Breast cancer (BC) is the most prevalent diagnosed cancer in women worldwide [1] and the second leading cause of death in women [2,3]. Although BC affects a large and growing population of women worldwide [2,3], the survival rates are fortunately on a steady rise mainly because of advancements in screening and treatment [4]. BC is associated with a reduced quality of life (QoL) because of its symptoms and treatment side effects [5]. BC symptoms encompass both physical and psychological impairments. Physical symptoms and sequelae include loss of power and function in limbs, lymphedema, muscle wasting, loss of bone density, chronic fatigue, pain, weight gain, and loss of appetite, whereas psychological symptoms include depression, anxiety associated with uncertainty, poor body image, loss of intimacy in relationships, reduced self-esteem, and cognitive dysfunction [6,7]. Scientific evidence has demonstrated that physical activity (PA) is the most well-established lifestyle factor associated with BC survival [8]. PA provides vital benefits to patients with BC and survivors of BC, including prevention of cancer recurrence; decreased side effects from treatment; and improvements in fitness, body size, and QoL [8,9]. Even women with advanced BC may benefit from moderate PA [10]. However, most of the aforementioned symptoms and treatment side effects are barriers to PA, which may present actual or perceived risks of injury or discomfort during physical exertion [11]. In addition, women with BC frequently report other barriers to PA such as lack of time and information [12]. In such circumstances, participation in and adherence to PA is low among survivors of BC [13]. Some studies have reported estimates of <10% of survivors of BC, which meet the PA guidelines and recommendations [14]. Overcoming these barriers to PA adherence among women with BC, who meet the current recommendations, is a complex challenge that requires innovative and engaging strategies. There is growing evidence regarding coaching interventions that effectively engage women with BC in PA [15]. These interventions are based on behavioral change theories (BCTs) such as the social cognitive theory [16], transtheoretical model [17], and self-determination theory [18]. Often, the implementation of these techniques is negatively affected by a lack of engagement of the users with the technology used for the delivery of the interventions. In that regard, feasibility studies such as the one described in this paper can provide insights into potential barriers to digital behavioral interventions.

Information and communication technologies enable cost-effective alternatives to help people reach PA recommendations through digital BCT-based interventions. In particular, advancements in mobile health (mHealth) technologies have increased interest in the research and development of mobile PA coaching systems and interventions [19]. Digital health transformation is also increasing this interest, especially in the current global situation because of the COVID-19 pandemic in which patients are encouraged to take a proactive approach to the self-management of their health and QoL. Mobile devices present unique capabilities that enable data collection in real-life scenarios [20,21], just-in-time behavioral information provision [22], and remote communication with health care professionals. These capabilities allow remote assessment, tracking, and monitoring in real-time and real-life environments, which form the basis of momentary ecological interventions [23]. As a result, these mHealth systems may potentially empower patients, promote behavior changes toward a healthier lifestyle, facilitate self-monitoring of symptoms and behaviors [24], provide real-time tailored support and motivation [21], improve their educational level [25], and allow patients the feeling of being in contact with their health care team [26].

mHealth coaching systems take advantage of these capabilities to implement functionalities and features based on BCTs to promote healthier behaviors. Among these systems, digital PA coaching interventions are well-received by women with BC, as suggested in the increasing body of scientific evidence [12,27]. However, to increase its acceptability among women with BC, it is essential that these digital persuasive systems are designed considering their contextual characteristics, needs, and preferences [28]. Each woman experiences her cancer journey in a particular and dynamic way, receiving different treatments and experiencing diverse symptoms such as fatigue or pain, which affect her emotional state, reduce her physical and cognitive capacities, and demand personalized social support. This unique experience requires that digital PA coaching systems consider not only their needs at the group level but also tailored to each individual [11,27]. Previous studies aimed at investigating the specific requirements of women with BC for digital PA coaching interventions have highlighted the importance of personalization in adapting these interventions to the specific individual’s conditions [12,27,29]. Lack of engagement and low perceived personal relevance of digital health systems are commonly associated with high levels of user abandonment [30]. In this sense, personalization can contribute to captivating and holding a person’s interest [31], resulting in an increased long-term engagement and adherence to these systems. In addition, personalization is associated with an increase in the effectiveness of BCT-based systems [32]. There is a lack of knowledge regarding the technology acceptance of real-time behavioral feedback using mHealth technologies in survivors of BC.

### Objective

This study presents the results of a small-scale evaluation of the acceptability of a personalized PA coaching mobile app for survivors of BC in the real world. The mobile app aimed to guide BC survivors on a plan to increase their PA, including behavioral and motivational aspects. The mobile solution also captured PA levels using a smartphone accelerometer. The objectives of this study were to (1) examine the potential acceptability or feasibility of the intervention; (2) assess usability and other aspects of the user experience; and (3) identify key considerations and aspects for future improvements, which may help increase and sustain acceptability and engagement.

## Methods

### Study Design

A mixed methods case series evaluation of usability and acceptability was conducted in this study. The use of a mixed methods design was chosen to capture both quantitative feedback about the mobile solution engagement and qualitative feedback about the user experience. This approach is well suited for understanding areas of improvement before designing larger studies. This study draws from the theoretical framework of acceptability (TFA) developed by Sekhon et al [32]. The TFA states that acceptability is influenced by 7 dimensions: affective attitude, burden, ethicality, intervention coherence, opportunity costs, perceived effectiveness, and self-efficacy. There are examples of the use of TFA in health technologies [33,34]. The objective of this study was to understand each participant’s perspective using both quantitative data (use of the solution) and qualitative data (interviews). A total of 4 women with BC participated in the study. The original plan was to recruit more participants; however, because of the COVID-19 pandemic, recruitment had to be halted.

The study comprised 3 sessions: initial, home, and final. In the initial session, a researcher provided participants with all the materials and instructions, installed the app on the smartphone, and explained the use of the system to the participants. In addition, data on participant characteristics were collected using questionnaires that covered demographics, technology use, and interests. In addition, 2 standardized scales were used: the Satisfaction with Life Scale [35] and the International Physical Activity Questionnaire–Short Form [36]. Satisfaction with Life Scale was used to capture health or life satisfaction as a potential mediator of overall engagement with the mobile solution.

Participants were then asked to use the app at home for a period of approximately 2 weeks in the home session, with data being collected from the app. App use and PA data were collected from the app and stored on a secure server during this period. The mobile solution was used to collect information on the PA and engagement of the users during this period. A period of 2 weeks was considered sufficient to capture the technology acceptance of the users. The 2 weeks were defined as sufficient time to identify major aspects related to acceptance of the solution, which was adapted to minimize disruptions in the clinical setting.

Finally, in the final session, participants returned materials to the research team that deleted any data stored on the smartphone and were asked to fill in 2 app evaluation scales and participate in a short individual interview. The participants completed the System Usability Scale [37] to assess the usability of the system. In addition, participants filled in the user version of the Mobile App Rating Scale [38] to assess the quality of the app. During the interviews, the interviewer provided trigger questions to the participants and took field notes. Questions used in the interviews were defined based on the TFA [32] and were built to cover various aspects of the participants’ user experience [39] associated with the different dimensions of acceptability and usability. The interviews were audio recorded, transcribed, and anonymized. The initial and final sessions took place in a private room at the Beacon Hospital headquarters, where only the session facilitator and the participant were present, and lasted 35 to 60 minutes. The data were collected from February to the beginning of March 2020.

### System and Materials

**Figure 1:**
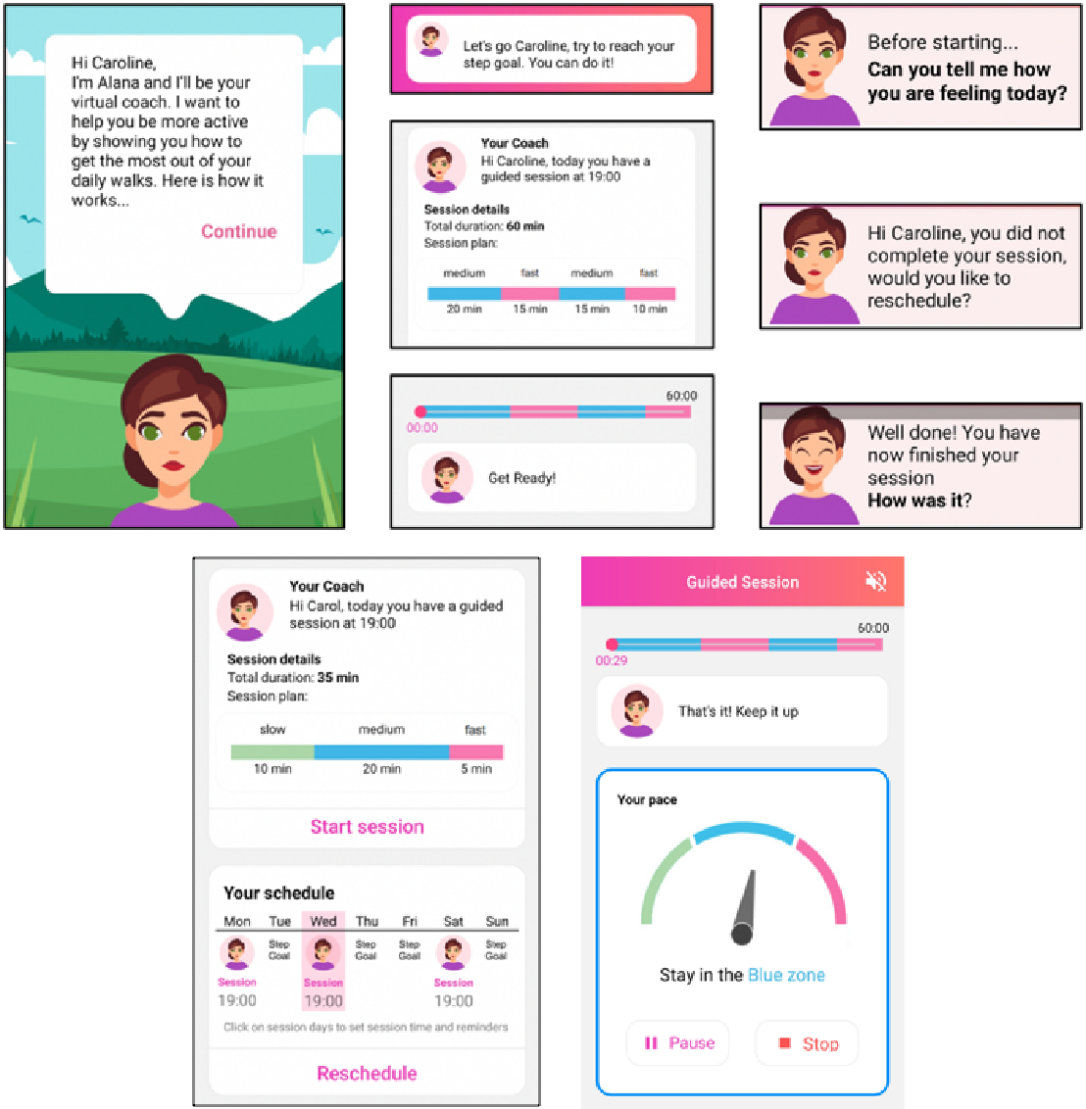
Screenshots of the developed mobile app.

The proposed system aimed to function as a mobile personal PA coach for survivors of BC and focused on optimizing walking activities to help them reach and maintain the levels of PA recommended in the guidelines. A user-centered design approach was followed to ensure that the end user needs were met. Relevant theories and evidence for successful PA interventions were used as the basis for the system design process. A detailed description of the design process is published in the studies by Monteiro-Guerra et al [12,40]. Screenshots of the developed mobile app are shown in Figure 1. The main features considered for the developed system prototype were as follows:

- A walking regimen
- Real-time activity monitoring and feedback
- Real-time guided sessions (with instructions to control session intensity)
- Adaptative training
- Personalized and encouraging communication
- Interface simulating an app-based coach
- Activity scheduling tool and reminders
- Activity history
- Weekly summary reports

The participants were provided with a waist pocket (Kalenji, Decathlon), wired in-ear headphones (Ear Pollution Bolt, iFrogz), and an Android smartphone (P Smart, Huawei). The smartphone was provided to ensure a similar user experience across participants. They also received printed instructions. The waist pocket was flexible and adjustable in size. The participants were instructed to wear the waist pocket and carry the smartphone while completing their activities. In addition, participants were asked to wear headphones to optimize the audio feedback delivery, providing a more private experience, especially when activities were performed in public places.

### Recruitment

The study was conducted in collaboration with the Beacon Hospital (Ireland). Participants were recruited by the specialized oncology team based on information collected from the Beacon Hospital patient database and were eligible to participate if they (1) were patients of oncology with a history of BC who had finished primary curative treatment (surgery, radiotherapy, and chemotherapy), (2) owned and used a mobile phone or smartphone, (3) were able to speak and read English, (4) had no known impairments or comorbidities, and (5) had no restrictions on PA. The recruitment process was performed in 2 rounds. First, an email, including the participant information leaflet, was sent to potential participants; subsequently, eligible participants were contacted by a phone call. Participants were required to provide informed consent before participation.

### Ethics Approval

This study was approved by the Research Ethics Committee of Beacon Hospital in Ireland (reference number: BEA0111) and received ethics exemption from the University College Dublin Human Research Ethics.

### Data Analysis

Participants’ PA levels were calculated based on their baseline session results, their mean number of steps per day, and the total number of sessions completed. Compliance with the training program was inferred considering three aspects: (1) the number of sessions completed of those proposed in the weekly plan, (2) the compliance with walking paces set in programmed sessions (equation 1), and (3) the number of nonsession days with the step goal achieved:

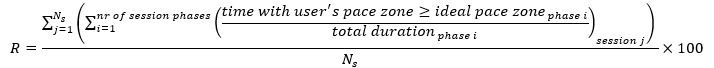



Here, *R* is the rate of compliance with the session format and *N_s_* is the total number of sessions completed.

Data from the standardized scales were analyzed based on their standard procedures. The International Physical Activity Questionnaire–Short Form results were also used to estimate the adherence of participants to PA guidelines [23].

A content analysis of the qualitative data collected in the interviews was conducted iteratively, ensuring that no critical information was overlooked. The findings of the interviews were discussed among the authors across these iterations. Several categories were defined using the app evaluation scales and interview data to outline the findings: (1) perceived impact or benefit, (2) positive feelings about system features and aesthetics, (3) usefulness, (4) intervention feasibility and appropriateness, (5) usability, and (6) suggested improvements. On the basis of the TFA constructs and usability aspects, relevant categories and subcategories were compiled, highlighting both the individual and common perspectives across the 4 participants. NVivo (version 12; QSR International) software was used for content analysis. Key illustrative quotes were selected to highlight the perspective of each participant.

## Results

### Participant Characteristics

Participant characteristics, including contextual details that may have influenced their experience with the system and intervention, are presented in Table 1. The age of the participants ranged from 35 to 61 years. Of the 4 participants, 3 (75%) were highly educated, and all 4 (100%) were at least somewhat skilled and interested in technology. Time since diagnosis ranged from approximately 1 to 3 years, and 50% (2/4) of participants reported having some cancer-related physical limitations. Approximately 50% (2/4) of participants worked in an office, whereas the other 50% (2/4) were housewives. All participants were moderately active; however, 50% (2/4) did not reach the recommended levels in the guidelines. Of the 4 participants, 1 (25%) was single and reported being slightly dissatisfied with her life.

**Table 1:** Participant characteristics.

Characteristics			P1		P2		P3		P4
**General characteristics**									
	Age (years)	61		43		35		54	
	Marital status	Married		Single		Married		Married	
	Education	Primary school		Postgraduate		Graduate		High school	
	Employment	Working (housewife)		Working (office)		Working (office+home)		Working (housewife)	
**Condition-related characteristics**									
	Date of diagnosis	February 2019		June 2018		October 2017		March 2018	
	Physical burdens	Fatigue and muscular pain		None reported		None reported		Lymphedema and joint pain	
**PA^a^ level**									
	IPAQ-SF^b^ score	Moderate		Moderate		Moderate		Moderate	
	Adherence to PA guidelines^c^	Not adherent		Adherent		Not adherent		Adherent	
	Sitting time (hours)	4		12		6		5	
	SWLS^d^ result	Highly satisfied		Slightly dissatisfied		Highly satisfied		Satisfied	
**Technology use, interest, and skill**									
	Smartphone use	High		High		High		High	
	Interest in mobile technologies	Somewhat interested		Somewhat interested		Interested		Somewhat interested	
	Self-reported skill with technology	Somewhat skilled		Skilled		Skilled		Somewhat skilled	
	“I like to experiment with new technology”^e^	Somewhat agree		Somewhat disagree		Agree		Somewhat agree	

^a^PA: physical activity.

^b^IPAQ-SF: International Physical Activity Questionnaire–Short Form.

^c^Adherence to >150 minutes per week=moderate activity or >75 minutes per week=vigorous activity, as inferred from the IPAQ-SF answers.

^d^SWLS: Satisfaction With Life Scale.

^e^Custom Likert scale made for the study.

### Acceptability

The participants mentioned that the app was nice, user-friendly, straightforward, and easy to understand. They found the app useful and had a positive perception that it presented a training schedule to remind them that they had a target to achieve:

I liked the system. I think it’s very well laid out.

P1

It’s a lovely, easy app to use.

P1

It was straightforward to use.

P2

I thought it was a really nice, use-friendly app; it is very straightforward, I think it is easy to understand.

P3

I really like the app itself...

P3

### Compliance

Training data on the initial level, intervention length, and compliance with the physical exercise program are shown in Table 2. One of the participants (P1) reported that she would have further followed the plan if it were not for bad weather and an ankle injury that she had experienced during the study period. Cold weather was also mentioned by P2. Regarding the training plan, some participants pointed out that some sessions were longer than the time they had available and that their current exercise habits were higher than what the app proposed:

On the first session of the week, [which is] 45 minutes, I would go, “Jesus, I only actually have time for 25 minute[s],” but you make the time for the 45 minutes and you feel good after it.

P3

**Table 2:** Intervention data overview.

Training aspect		P1	P2	P3	P4
Intervention length (weeks)		3	2	3	2.5
Total number of planned sessions, N		8	7	9	8
Total number of completed sessions, n (%)^a^		5 (63)	7 (100)	7 (78)	7 (88)
Daily step count, mean (SD)^b^		8251.7 (2742.9)	6838.3 (2335.9)	6377.4 (1942.3)	6292.4 (3575.8)
**Training compliance**					
	With guided sessions (%)^a^	62.5	100	77.8	87.5
	With step daily goal (%)^b^	66.7	28.6	70	60
Compliance with session format, mean (SD)^b^		97.7 (1.5)	98.6 (1.0)	95.0 (4.2)	87.8 (9.4)

^a^Includes sessions where the full data set was not recorded; however, participants reported having completed the session.

^b^Results calculated from sessions in which data were properly collected and stored for the duration of the session.

### Usability

The results of the scores for the System Usability Scale and the user version of the Mobile Application Rating Scale are presented in Table 3. Participants mentioned that the technical difficulties with the system data collection, particularly with the miscount of steps, could make them feel frustrated. The system was somewhat cumbersome to use, given the need to use an extra phone for the study and the difficulty in carrying the phone while wearing the headphones:

If it was on my iPhone, I probably wouldn’t think twice about using it.

P4

No, just bringing the (extra) phone with me. That’s all.

P4

I put it in my pocket—it was grand. I always have zip pockets anyway. It was just in my pocket.

P4

**Table 3:** System Usability Scale (SUS) scores out of 100 and the mean user version of the Mobile App Rating Scale (uMARS) scores out of 5.

App evaluation scales or aspects			P1		P2		P3		P4
SUS			72.5		77.5		95		80
**uMARS,** **mean (SD)**									
	Engagement	4 (0.7)		4 (0.7)		3.8 (1.3)		2.6 (1.1)	
	Functionality	3.8 (1.5)		4.5 (0.6)		4.75 (0.5)		4 (0.8)	
	Aesthetics	4.7 (0.5)		4.3 (0.6)		4.7 (0.6)		3.7 (0.6)	
	Information	3.8 (0.5)		4.5 (0.6)		4.5 (0.6)		4.3 (1.0)	
	Overall quality	4.0 (0.4)		4.3 (0.2)		4.4 (0.4)		3.6 (0.7)	
	App subjective quality	4.3 (1.0)		3.8 (0.5)		4.0 (0.8)		3.3 (1.7)	
	Perceived impact	4.7 (0.5)		3.0 (0.9)		4.0 (0.6)		3.5 (0.8)	

About the functionalities of the app, the participants highlighted the recognition of achievements and the possibility to check their activity history and found that the rescheduling option was *excellent*. The system made them more aware and conscious of being active and stimulated them to be more active. Moreover, participants felt that they were not on their own. Guided sessions’ cues to slow down and speed up and the progress in time through the session was important to encourage them to keep going, creating a feeling of satisfaction when they followed what the plan proposed:

I enjoyed it and I loved the fact that I could reschedule, because I could work it around the days and the times that suited me. The reschedule and the retiming I found excellent.

P1

I liked when she told you to speed up or slow down.

P2

I liked that she spoke to you and said, “We are now moving into this.”

P2

You’d know—she’d say, “Five minutes more” or whatever—and that was good. I can imagine that’s very encouraging. If I was running and she had been saying those things to me, I would have been encouraged to keep going. She’d say, “Only a few more minutes, or you’re going faster than you need to.” Yes.

P2

Rescheduling functionality was perceived to be very useful. Participants also had very positive perceptions toward being able to check their activity progress during the day and their past activity history, which they also found useful.

Finally, when questioned about battery consumption during the sessions, the participants stated that they did not perceive that it drained the battery.

### Potential Improvements

Participants also suggested several improvements that they believed would make the system fit their preferences more effectively. These involved the inclusion of other health aspects, such as sleep and diet, in a diary to compare with the PA progress; combining the guided exercise with music, the possibility to pair the system with a wearable, receiving feedback on the sessions, and checking the number of steps taken; and that the pausing function should be easier, avoiding taking the phone out and pausing it at the traffic lights.

Participants also suggested that it would be nice to have more options to customize the training plan and sessions, with long-term goals (eg, to do a 5 km run), being able to customize the session distance or duration, or adding more sessions in a week and having the option to customize the voice of the coach. They also mentioned the idea of relating the pace zones with the heart rate to allow the user to customize the training plan and have a cooldown period in the guided sessions.

Regarding the history functionality, suggested improvements included having detailed information of all the sessions in the week, including information on the session format from future sessions in the week (eg, information on duration), allowing the checking of activity counts from previous days on the MyActivity screen, integrating the option to manually add activities for previous days, and considering how to share their data with the health care professional.

Finally, participants also said that all functionalities and guided sessions made them think that a lot of work had been put into the app and that they believed it was safe and was going to support them, which created a feeling of trust:

[About the guided sessions] I liked how often she came back in, because when your phone is in your pocket, sometimes it is like, “Oh God, is it still working, or has my phone shut down the app, and is it still tracking me?,” but she comes in so often that it is reassuring—you don’t have to keep taking your phone out to make sure that it is working.

P3

I really liked that it asked you how you felt at the end of it. I really like at the start the way it asks you how you are feeling and if there is [any] bad weather and stuff like that—I think that was really good. You feel safe while you are using it—you feel like it is going to be accurate. You feel like it is supporting you.

P3

It just feels like you have thought of everything with this, so you feel like you are in safe hands...It is nice to have the voice in the ears to motivate you.

P3

I think there was one day I was very tired. I suffer a bit from insomnia anyway...I was very tired when I finished. Whether it was my imagination...the session seemed to adapt to that.

P4

### Potential Benefits

Participants indicated that the app had the potential to work well for survivors of BC as an incentive, not just because of the walks but also as it allows the users to check their progress, which they believed was motivating. It could be beneficial at different stages during the treatment, particularly in those who have never done any exercise before or who are trying to get back into it after treatment:

I think it would be quite high [the potential benefit], I really do. I would imagine [...] at least 60/65 percent [engaged], if not higher, [for it to] benefit.

P1

[...] for the first eight weeks at least after your radiotherapy [means] you’re tired, you’re raw, and you’re scared. I think getting out with the app and getting walking at that point would be good, both physically and mentally.

P1

I think it could make an absolute brilliant overall meaningful exercise and moving forward positively.

P1

I think it would work really well, yes.

P2

Because it is the incentive that they have, it isn’t just about going for a walk—it’s going for a walk, but you can track your progress, which I think is quite motivating to say. [...] Yes, just different stages during treatment I think could be very beneficial, yes, or particularly patients where they haven’t done any exercise and now, they’re trying to get back into it. It is motivating, it is encouraging to say that it’s there.

P2

I think it would really help people. When you finish treatment, you don’t know where to go, you don’t know what to do, [or] what is safe to do—you question everything. I think to have something like this on your phone just for you, [to] set yourself your targets, get out, and do it—I think it would be so beneficial for so many people.

P3

I think that the target of three a week is really good because you know that is what you have to do. I think the step tracker is so great for people who don’t have a smart watch. Giving you information like that can only be beneficial to your health.

P3

## Discussion

### Principal Findings

This study evaluated the potential acceptability and several aspects of user experience of mobile-based interventions for survivors of BC. A mixed methods case series study design was used to provide a deeper understanding of the individual experiences of participants. The findings cover aspects associated with the feasibility of the PA intervention, affective attitude toward the system, coherence and usability, system burden, perceived impact and quality, and potential effectiveness.

Overall, the participants found the system friendly and easy to use and showed a very positive attitude toward its various system features and aesthetics. Participants’ scores on the usability and quality scales were good and seemed consistent. They perceived that the system was encouraging, increased their consciousness of their PA, and pushed them to go out more, which induced positive feelings. Furthermore, the participants had positive opinions toward the guided sessions, looking at their activity progress and history and being recognized for their achievements. All participants found it feasible and fairly easy to integrate into their daily lives and had very positive perspectives on having defined goals and being able to reschedule sessions.

This study reinforced the advantages of following user-centered design approaches and involving users at different design stages of the product. The findings from this study will inform the development of the next iteration of the system to maximize usability and the potential acceptability across a wider range of survivors of BC, which may improve the future success of the system [41]. We believe that the results of this preliminary evaluation point to the potential of the tool proposed in this thesis to support PA in survivors of BC. In this study, several challenges related to user-centered design emerged. The study has to be conducted in a way to minimize clinical disruptions, and consequently, the time for follow-up (eg, flexible duration of the intervention) was decided to minimize the burden on patients and clinicians (eg, not setting up study visits that are not aligned with the clinical visits). We also decided to provide smartphones to minimize issues with some patients using older phones with limited capacity and performance. This might also pose a challenge in the sense that some users might not have been familiarized with the provided smartphones. Overall, this preliminary feasibility study should always balance the need to be as close as possible to the real world and the potential minimization of bias in the study.

### Potential Acceptability and Feasibility

Step count monitoring leads to short- and long-term step count increases [42], and in our study, participants mentioned being conscious of the step goals but not following them every day, which is reflected in lower compliance with that aspect of training in all participants. This can also be because of the need to use an extra phone for the study, which seemed to be slightly challenging, as they needed some time to get used to the different operating system and sometimes left it at home. Nevertheless, the strategy for using the extra phone was to obtain more accurate feedback on the system measurements. The code provides a basic step tracking tool implementation using the Android accelerometer signal and a basic peak detection algorithm [43] to detect when the user takes a step.

The participants seemed to agree on the feasibility of the training plan and the format of the sessions, with the different phases at a certain pace. In addition, they liked having a plan and clear targets and did not seem to feel it was overprescriptive. Participants liked the format of the sessions and that it was different for each session, and they found it positive that the sessions pushed them physically. The app adapted to the session’s difficulty when patients reported feeling tired, which was perceived positively and reinforced the importance of the adaptive training functionality.

These aspects highlight the importance of involving health professionals in the design process of digital tools [44]. However, the participants had some suggestions for improvement regarding the PA plan. They argued that it would be preferable if the sessions ended with a cooling down phase. Although there are many proposed benefits of an active cooldown compared with a passive cooldown, only a few of these benefits are supported by research. However, most individuals perceived an active cooldown to be more beneficial than a passive cooldown [45], and it is important to consider it in future exercise session design. Other customizations of the PA plan, such as adding more sessions, must also be discussed individually. Nevertheless, there are specific prescription guidelines [46] that are a good practice to be followed to enhance patient safety.

Participants mentioned the challenge of keeping with the plan when it was cold or rainy. In particular, unfit adults tend not to participate in PA when the weather is unpleasant [47]. This highlights the importance of exploring ways in which the system considers and adapts to the users’ context (eg, location and weather).

System usability is a critical aspect associated with intervention coherence and influences self-efficacy, which are 2 important aspects of acceptability [48]. A major usability issue revealed in the study was the poor accessibility of the weekly summary report, which was somewhat *hidden* in the history tab and, therefore, limited the participants’ use of this feature. Despite these issues, the participants were confident in using the system on their own and highlighted the importance of the initial demonstration in the first study session and of trying out the app for a while independently. The participants gave some scores close to the maximum of 5 in aesthetics and functionality, and there were some mixed scores among the 4 participants for entertainment, target group, customization, and quantity of information. Related works suggest that these aspects may be associated with system engagement [49].

From the interview data, participants seemed to agree that the app was very positive, user-friendly, useful, easy to use, and motivating. All the functionalities were perceived as very useful by at least one of the participants. The main component of the system, the guided sessions, had very positive opinions from all the participants. Commenting on the coach’s communication during the session, participants suggested that having the voice with the cues about pace, progress, and encouragement while doing an activity was *lovely*. There is already solid scientific literature demonstrating the potential of digital health interventions, particularly when combining PA monitors, tailored motivational messaging, and web-based coaching, in increasing PA and having the potential to improve health outcomes [50].

The participants also reported that they felt good when they completed the sessions. Scientific research indicates that exercise is associated with positive mood changes, even when physiological benefits are not found [51]. This before and after exercise mood and fatigue feedback seems to be very important to possibly routine the adjustment of durations and intensities, which increasingly facilitates positive postexercise feelings and better maintenance of exercise [52].

With regard to personal data sharing, participants mentioned being open to sharing their personal data in exchange for a more personalized approach, which is in line with previous literature [12]. This attitude may be associated with a feeling of trust in the app, given that this was a scientific research study using an evidence-based app and validated by a health care professional (eg, oncologist and specialist nurse).

### Future Considerations and Improvements

Participants identified some technical inconsistencies and provided helpful suggestions on how the mobile solution improved, which may influence the acceptability and feasibility of the system and demonstrates the importance of system evaluation with users at an early stage [53,54].

To our knowledge, there are few mobile app–based PA interventions, specifically designed for survivors of BC, that have been submitted for some type of evaluation [11,55-58]. Similarly, their findings point to the potential interest of these individuals toward a PA app, and among the successful and useful features were the balanced exercise program, visual support, viewing personal progress, activity reminders, and acknowledgment of activity achievements. However, in accordance with our findings, participants in that study perceived the exercises as being too easy to perform overall and wanted to feel more challenged. In addition, they wanted further adaptability from the app, for example, in learning from their daily routine to adjust communication. In this sense, Marcu et al [11] suggested the potential for adaptability and customization of features to increase system effectiveness. This is in line with our findings and supports the continued improvement of the system proposed in this thesis and further exploration of modules for personalized communication and adaptive PA prescription.

Overall, this study shows some promising results regarding the concept proposed for a PA coaching system for survivors of BC. Considering the aspects discussed here, an improved version of this system may have the potential to be accepted and engage these individuals, which may ultimately lead to an increase in PA adherence. Future work is required to assess and optimize the reliability of the monitoring and activity prescription systems, improve the motivational and personalization functionalities used, and assess the feasibility of the system in the long term. Only after these stages should the final step—the efficacy evaluation of the system in a rigorous trial—be considered.

### Limitations

A limitation of this study is the small number of participants and also the short duration of the intervention, considering that survivors of BC do require support for long periods. This work is an early evaluation to gather preliminary insights from end users’ perspectives on the concept and inform future app versions and intervention improvements. Future studies should test the acceptability and feasibility of the intervention with a higher number of participants and for longer periods before conducting more controlled trials.

The study protocol did not address the collection of data regarding recruitment (eg, the number of patients who received and opened the email with the invitation and the response rate of invitation by phone calls). This information may be highly relevant for the designing of larger studies.

All 4 participants had high levels of education and digital literacy and had at least some experience using mobile apps. In addition, all were from the same private hospital, which may be associated with an affluent socioeconomic background, better care, high education, and high awareness of the importance of self-management (eg, PA). Furthermore, the participants were moderately active and between 1 and 3 years after the main treatment. Therefore, the sample may not be representative of the wider population. Future work should consider a larger and more diverse sample of participants considering, for example, digital literacy, PA awareness and levels, the type of care, and the number of years since treatment.

Overall, PA is important for patients with BC; however, it also includes the use of resistance exercise, which was not included in this digital intervention. Future work and research in this area should also include resistance exercises, and we should consider that the sample of patients that participated in this study might not be generalizable to the general population. Our participants were moderately active and highly educated, and future research should explore how transferable the results can be to other cohorts of patients.

### Conclusions

The participants of this study showed high usability and satisfaction. Further research should look into larger and more diverse cohorts and may have the potential to be acceptable and feasible for survivors of BC, particularly those in the early stages of survivorship. However, functional improvements and additional coaching content (eg, other activity types) should be considered in future iterations of the concept to be appropriate for a wider population of survivors of BC. Furthermore, more work is needed to expand system customization and automatic personalization to provide content adjusted to their individual needs and preferences at each stage in their survivorship journey.

Overall, the early results presented in this study point to the potential of this tool concept to provide a friendly and satisfying coaching experience to the users, which may help improve PA adherence in survivors of BC. Therefore, this study supports future work on improving and evaluating the proposed system. Following the resolution of the technical issues experienced in this study, future evaluations of the system are needed to assess system acceptability and feasibility with a larger and more varied sample and for more prolonged periods to evaluate the system's potential for engagement and assess the influence of motivational and personalization strategies in sustaining system adherence.

## Abbreviations

BC: breast cancer
BCT: behavioral change theory
mHealth: mobile health
PA: physical activity
QoL: quality of life
TFA: theoretical framework of acceptability
